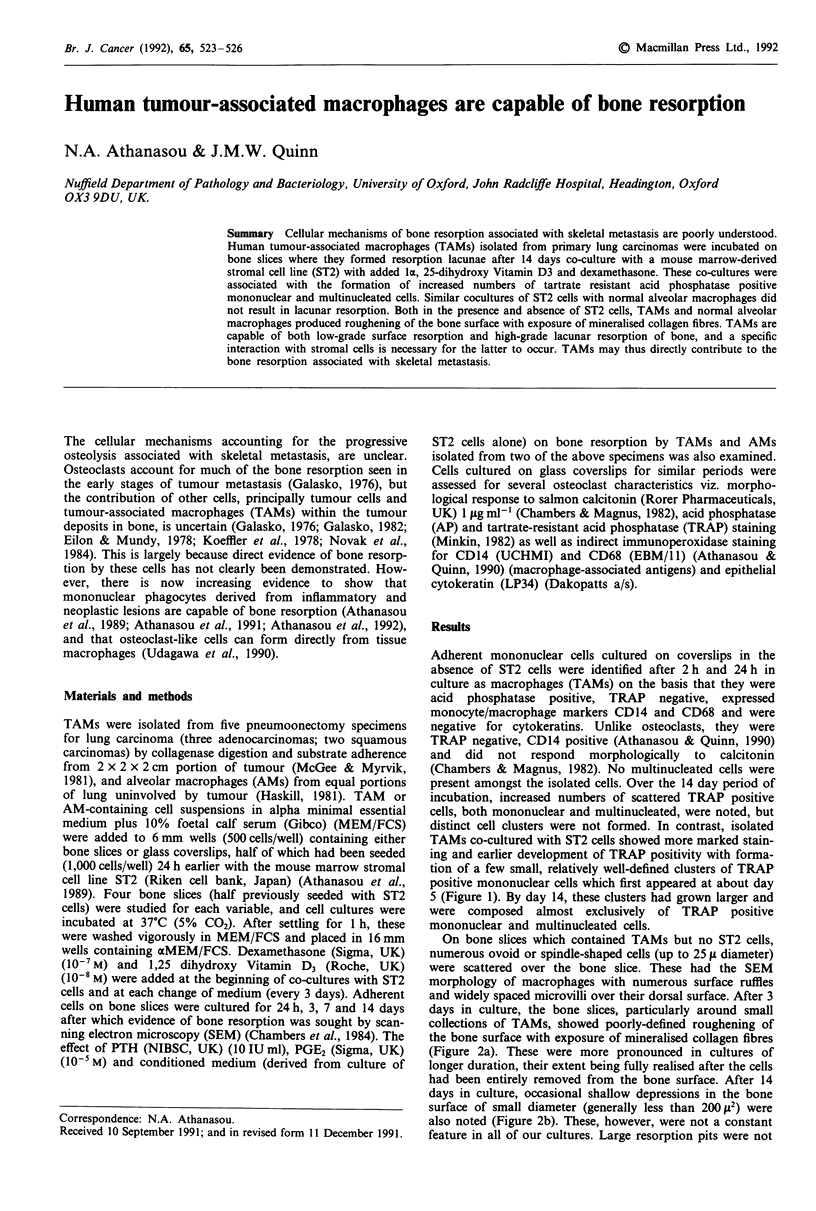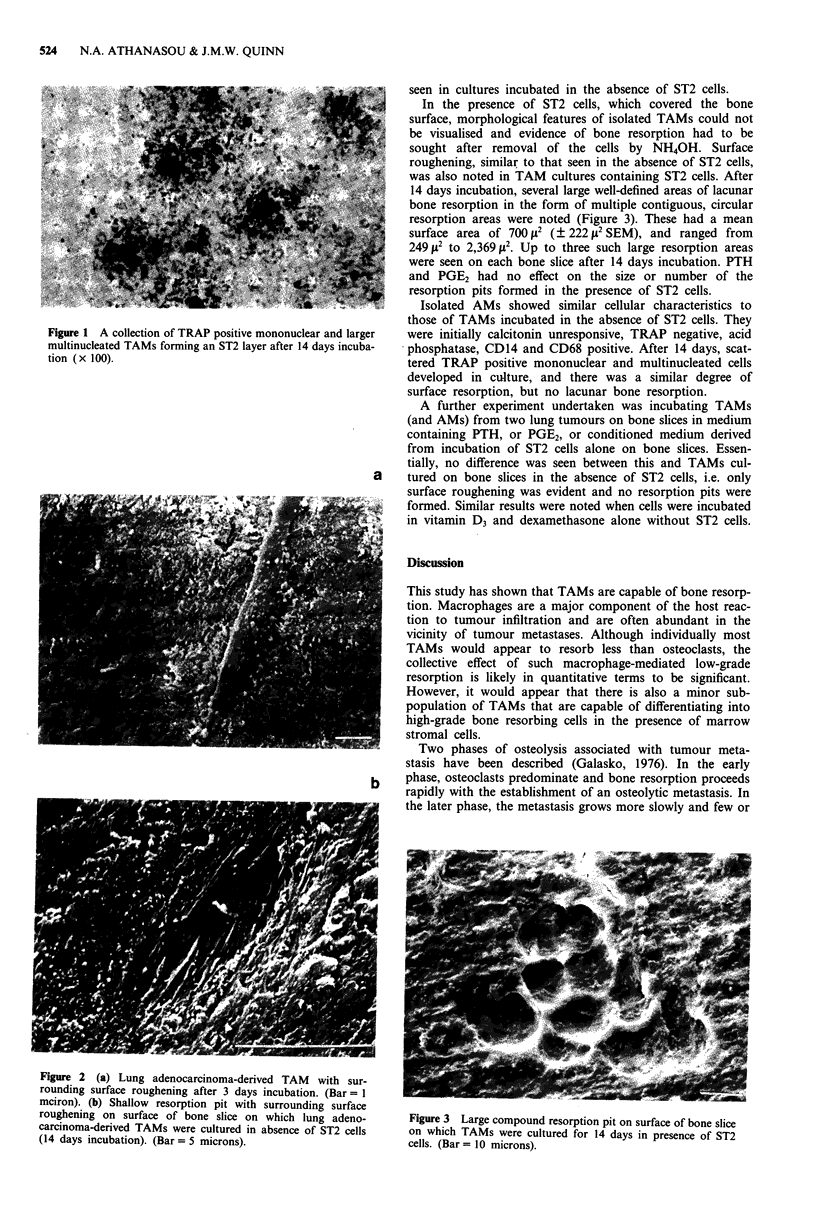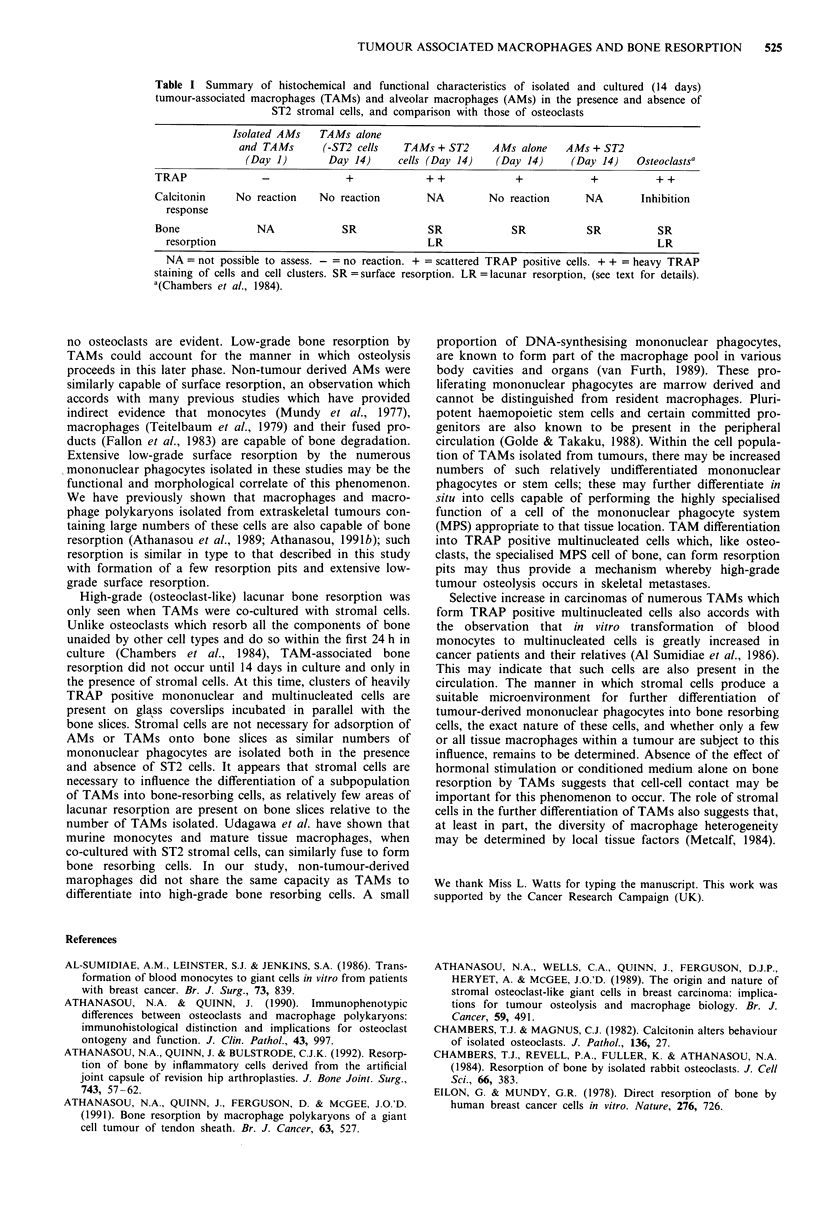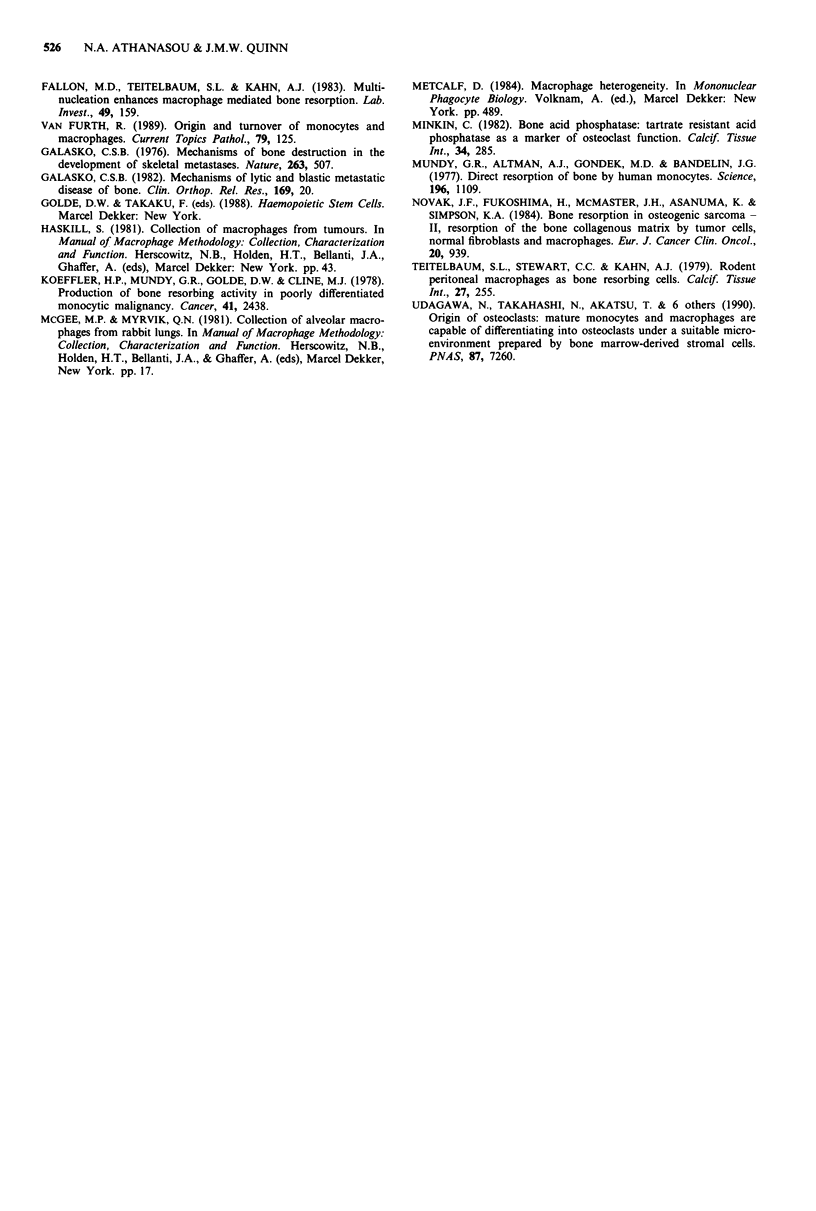# Human tumour-associated macrophages are capable of bone resorption.

**DOI:** 10.1038/bjc.1992.108

**Published:** 1992-04

**Authors:** N. A. Athanasou, J. M. Quinn

**Affiliations:** Nuffield Department of Pathology and Bacteriology, University of Oxford, John Radcliffe Hospital, Headington, UK.

## Abstract

**Images:**


Br. J. Cancer (1992), 65, 523-526 © Macmillan Press Ltd., 1992

### Human tumour-associated macrophages are capable of bone resorption

N.A. Athanasou & J.M.W. Quinn

*Nuffield Department of Pathology and Bacteriology, University of Oxford, John Radcliffe Hospital, Headington, Oxford OX3 9DU, UK.*

**Summary** Cellular mechanisms of bone resorption associated with skeletal metastasis are poorly understood. Human tumour-associated macrophages (TAMs) isolated from primary lung carcinomas were incubated on bone slices where they formed resorption lacunae after 14 days co-culture with a mouse marrow-derived stromal cell line (ST2) with added 1α, 25-dihydroxy Vitamin D3 and dexamethasone. These co-cultures were associated with the formation of increased numbers of tartrate resistant acid phosphatase positive mononuclear and multinucleated cells. Similar cocultures of ST2 cells with normal alveolar macrophages did not result in lacunar resorption. Both in the presence and absence of ST2 cells, TAMs and normal alveolar macrophages produced roughening of the bone surface with exposure of mineralised collagen fibres. TAMs are capable of both low-grade surface resorption and high-grade lacunar resorption of bone, and a specific interaction with stromal cells is necessary for the latter to occur. TAMs may thus directly contribute to the bone resorption associated with skeletal metastasis.

The cellular mechanisms accounting for the progressive osteolysis associated with skeletal metastasis, are unclear. Osteoclasts account for much of the bone resorption seen in the early stages of tumour metastasis (Galasko, 1976), but the contribution of other cells, principally tumour cells and tumour-associated macrophages (TAMs) within the tumour deposits in bone, is uncertain (Galasko, 1976; Galasko, 1982; Eilon & Mundy, 1978; Koeffler *et al.*, 1978; Novak *et al.*, 1984). This is largely because direct evidence of bone resorption by these cells has not clearly been demonstrated. However, there is now increasing evidence to show that mononuclear phagocytes derived from inflammatory and neoplastic lesions are capable of bone resorption (Athanasou *et al.*, 1989; Athanasou *et al.*, 1991; Athanasou *et al.*, 1992), and that osteoclast-like cells can form directly from tissue macrophages (Udagawa *et al.*, 1990).

### Materials and methods

TAMs were isolated from five pneumoonectomy specimens for lung carcinoma (three adenocarcinomas; two squamous carcinomas) by collagenase digestion and substrate adherence from 2 × 2 × 2 cm portion of tumour (McGee & Myrvik, 1981), and alveolar macrophages (AMs) from equal portions of lung uninvolved by tumour (Haskill, 1981). TAM or AM-containing cell suspensions in alpha minimal essential medium plus 10% foetal calf serum (Gibco) (MEM/FCS) were added to 6 mm wells (500 cells/well) containing either bone slices or glass coverslips, half of which had been seeded (1,000 cells/well) 24 h earlier with the mouse marrow stromal cell line ST2 (Riken cell bank, Japan) (Athanasou *et al.*, 1989). Four bone slices (half previously seeded with ST2 cells) were studied for each variable, and cell cultures were incubated at 37°C (5% $CO_2$). After settling for 1 h, these were washed vigorously in MEM/FCS and placed in 16 mm wells containing αMEM/FCS. Dexamethasone (Sigma, UK) ($10^{-7}$ M) and 1,25 dihydroxy Vitamin $D_3$ (Roche, UK) ($10^{-8}$ M) were added at the beginning of co-cultures with ST2 cells and at each change of medium (every 3 days). Adherent cells on bone slices were cultured for 24 h, 3, 7 and 14 days after which evidence of bone resorption was sought by scanning electron microscopy (SEM) (Chambers *et al.*, 1984). The effect of PTH (NIBSC, UK) (10 IU ml), $PGE_2$ (Sigma, UK) ($10^{-5}$ M) and conditioned medium (derived from culture of ST2 cells alone) on bone resorption by TAMs and AMs isolated from two of the above specimens was also examined. Cells cultured on glass coverslips for similar periods were assessed for several osteoclast characteristics viz. morphological response to salmon calcitonin (Rorer Pharmaceuticals, UK) 1 $\mu g\ ml^{-1}$ (Chambers & Magnus, 1982), acid phosphatase (AP) and tartrate-resistant acid phosphatase (TRAP) staining (Minkin, 1982) as well as indirect immunoperoxidase staining for CD14 (UCHMI) and CD68 (EBM/11) (Athanasou & Quinn, 1990) (macrophage-associated antigens) and epithelial cytokeratin (LP34) (Dakopatts a/s).

Correspondence: N.A. Athanasou.
Received 10 September 1991; and in revised form 11 December 1991.

### Results

Adherent mononuclear cells cultured on coverslips in the absence of ST2 cells were identified after 2 h and 24 h in culture as macrophages (TAMs) on the basis that they were acid phosphatase positive, TRAP negative, expressed monocyte/macrophage markers CD14 and CD68 and were negative for cytokeratins. Unlike osteoclasts, they were TRAP negative, CD14 positive (Athanasou & Quinn, 1990) and did not respond morphologically to calcitonin (Chambers & Magnus, 1982). No multinucleated cells were present amongst the isolated cells. Over the 14 day period of incubation, increased numbers of scattered TRAP positive cells, both mononuclear and multinucleated, were noted, but distinct cell clusters were not formed. In contrast, isolated TAMs co-cultured with ST2 cells showed more marked staining and earlier development of TRAP positivity with formation of a few small, relatively well-defined clusters of TRAP positive mononuclear cells which first appeared at about day 5 (Figure 1). By day 14, these clusters had grown larger and were composed almost exclusively of TRAP positive mononuclear and multinucleated cells.

On bone slices which contained TAMs but no ST2 cells, numerous ovoid or spindle-shaped cells (up to 25 μ diameter) were scattered over the bone slice. These had the SEM morphology of macrophages with numerous surface ruffles and widely spaced microvilli over their dorsal surface. After 3 days in culture, the bone slices, particularly around small collections of TAMs, showed poorly-defined roughening of the bone surface with exposure of mineralised collagen fibres (Figure 2a). These were more pronounced in cultures of longer duration, their extent being fully realised after the cells had been entirely removed from the bone surface. After 14 days in culture, occasional shallow depressions in the bone surface of small diameter (generally less than 200 $\mu^2$) were also noted (Figure 2b). These, however, were not a constant feature in all of our cultures. Large resorption pits were not

**Figure 1** A collection of TRAP positive mononuclear and larger multinucleated TAMs forming an ST2 layer after 14 days incubation (× 100).

**Figure 2** (**a**) Lung adenocarcinoma-derived TAM with surrounding surface roughening after 3 days incubation. (Bar = 1 mciron). (**b**) Shallow resorption pit with surrounding surface roughening on surface of bone slice on which lung adenocarcinoma-derived TAMs were cultured in absence of ST2 cells (14 days incubation). (Bar = 5 microns).

seen in cultures incubated in the absence of ST2 cells.

In the presence of ST2 cells, which covered the bone surface, morphological features of isolated TAMs could not be visualised and evidence of bone resorption had to be sought after removal of the cells by $NH_4OH$. Surface roughening, similar to that seen in the absence of ST2 cells, was also noted in TAM cultures containing ST2 cells. After 14 days incubation, several large well-defined areas of lacunar bone resorption in the form of multiple contiguous, circular resorption areas were noted (Figure 3). These had a mean surface area of $700\,\mu^2$ ($\pm 222\,\mu^2$ SEM), and ranged from $249\,\mu^2$ to $2{,}369\,\mu^2$. Up to three such large resorption areas were seen on each bone slice after 14 days incubation. PTH and $PGE_2$ had no effect on the size or number of the resorption pits formed in the presence of ST2 cells.

Isolated AMs showed similar cellular characteristics to those of TAMs incubated in the absence of ST2 cells. They were initially calcitonin unresponsive, TRAP negative, acid phosphatase, CD14 and CD68 positive. After 14 days, scattered TRAP positive mononuclear and multinucleated cells developed in culture, and there was a similar degree of surface resorption, but no lacunar bone resorption.

A further experiment undertaken was incubating TAMs (and AMs) from two lung tumours on bone slices in medium containing PTH, or $PGE_2$, or conditioned medium derived from incubation of ST2 cells alone on bone slices. Essentially, no difference was seen between this and TAMs cultured on bone slices in the absence of ST2 cells, i.e. only surface roughening was evident and no resorption pits were formed. Similar results were noted when cells were incubated in vitamin $D_3$ and dexamethasone alone without ST2 cells.

### Discussion

This study has shown that TAMs are capable of bone resorption. Macrophages are a major component of the host reaction to tumour infiltration and are often abundant in the vicinity of tumour metastases. Although individually most TAMs would appear to resorb less than osteoclasts, the collective effect of such macrophage-mediated low-grade resorption is likely in quantitative terms to be significant. However, it would appear that there is also a minor subpopulation of TAMs that are capable of differentiating into high-grade bone resorbing cells in the presence of marrow stromal cells.

Two phases of osteolysis associated with tumour metastasis have been described (Galasko, 1976). In the early phase, osteoclasts predominate and bone resorption proceeds rapidly with the establishment of an osteolytic metastasis. In the later phase, the metastasis grows more slowly and few or

**Figure 3** Large compound resorption pit on surface of bone slice on which TAMs were cultured for 14 days in presence of ST2 cells. (Bar = 10 microns).

**Table I** Summary of histochemical and functional characteristics of isolated and cultured (14 days) tumour-associated macrophages (TAMs) and alveolar macrophages (AMs) in the presence and absence of ST2 stromal cells, and comparison with those of osteoclasts

| | *Isolated AMs and TAMs (Day 1)* | *TAMs alone (-ST2 cells Day 14)* | *TAMs + ST2 cells (Day 14)* | *AMs alone (Day 14)* | *AMs + ST2 (Day 14)* | *Osteoclasts*[a] |
|---|---|---|---|---|---|---|
| TRAP | – | + | ++ | + | + | ++ |
| Calcitonin response | No reaction | No reaction | NA | No reaction | NA | Inhibition |
| Bone resorption | NA | SR | SR LR | SR | SR | SR LR |

NA = not possible to assess. – = no reaction. + = scattered TRAP positive cells. ++ = heavy TRAP staining of cells and cell clusters. SR = surface resorption. LR = lacunar resorption, (see text for details). [a](Chambers *et al.*, 1984).

no osteoclasts are evident. Low-grade bone resorption by TAMs could account for the manner in which osteolysis proceeds in this later phase. Non-tumour derived AMs were similarly capable of surface resorption, an observation which accords with many previous studies which have provided indirect evidence that monocytes (Mundy *et al.*, 1977), macrophages (Teitelbaum *et al.*, 1979) and their fused products (Fallon *et al.*, 1983) are capable of bone degradation. Extensive low-grade surface resorption by the numerous mononuclear phagocytes isolated in these studies may be the functional and morphological correlate of this phenomenon. We have previously shown that macrophages and macrophage polykaryons isolated from extraskeletal tumours containing large numbers of these cells are also capable of bone resorption (Athanasou *et al.*, 1989; Athanasou, 1991*b*); such resorption is similar in type to that described in this study with formation of a few resorption pits and extensive low-grade surface resorption.

High-grade (osteoclast-like) lacunar bone resorption was only seen when TAMs were co-cultured with stromal cells. Unlike osteoclasts which resorb all the components of bone unaided by other cell types and do so within the first 24 h in culture (Chambers *et al.*, 1984), TAM-associated bone resorption did not occur until 14 days in culture and only in the presence of stromal cells. At this time, clusters of heavily TRAP positive mononuclear and multinucleated cells are present on glass coverslips incubated in parallel with the bone slices. Stromal cells are not necessary for adsorption of AMs or TAMs onto bone slices as similar numbers of mononuclear phagocytes are isolated both in the presence and absence of ST2 cells. It appears that stromal cells are necessary to influence the differentiation of a subpopulation of TAMs into bone-resorbing cells, as relatively few areas of lacunar resorption are present on bone slices relative to the number of TAMs isolated. Udagawa *et al.* have shown that murine monocytes and mature tissue macrophages, when co-cultured with ST2 stromal cells, can similarly fuse to form bone resorbing cells. In our study, non-tumour-derived marophages did not share the same capacity as TAMs to differentiate into high-grade bone resorbing cells. A small proportion of DNA-synthesising mononuclear phagocytes, are known to form part of the macrophage pool in various body cavities and organs (van Furth, 1989). These proliferating mononuclear phagocytes are marrow derived and cannot be distinguished from resident macrophages. Pluripotent haemopoietic stem cells and certain committed progenitors are also known to be present in the peripheral circulation (Golde & Takaku, 1988). Within the cell population of TAMs isolated from tumours, there may be increased numbers of such relatively undifferentiated mononuclear phagocytes or stem cells; these may further differentiate *in situ* into cells capable of performing the highly specialised function of a cell of the mononuclear phagocyte system (MPS) appropriate to that tissue location. TAM differentiation into TRAP positive multinucleated cells which, like osteoclasts, the specialised MPS cell of bone, can form resorption pits may thus provide a mechanism whereby high-grade tumour osteolysis occurs in skeletal metastases.

Selective increase in carcinomas of numerous TAMs which form TRAP positive multinucleated cells also accords with the observation that *in vitro* transformation of blood monocytes to multinucleated cells is greatly increased in cancer patients and their relatives (Al Sumidiae *et al.*, 1986). This may indicate that such cells are also present in the circulation. The manner in which stromal cells produce a suitable microenvironment for further differentiation of tumour-derived mononuclear phagocytes into bone resorbing cells, the exact nature of these cells, and whether only a few or all tissue macrophages within a tumour are subject to this influence, remains to be determined. Absence of the effect of hormonal stimulation or conditioned medium alone on bone resorption by TAMs suggests that cell-cell contact may be important for this phenomenon to occur. The role of stromal cells in the further differentiation of TAMs also suggests that, at least in part, the diversity of macrophage heterogeneity may be determined by local tissue factors (Metcalf, 1984).

We thank Miss L. Watts for typing the manuscript. This work was supported by the Cancer Research Campaign (UK).

### References

AL-SUMIDIAE, A.M., LEINSTER, S.J. & JENKINS, S.A. (1986). Transformation of blood monocytes to giant cells *in vitro* from patients with breast cancer. *Br. J. Surg.*, **73**, 839.

ATHANASOU, N.A. & QUINN, J. (1990). Immunophenotypic differences between osteoclasts and macrophage polykaryons: immunohistological distinction and implications for osteoclast ontogeny and function. *J. Clin. Pathol.*, **43**, 997.

ATHANASOU, N.A., QUINN, J. & BULSTRODE, C.J.K. (1992). Resorption of bone by inflammatory cells derived from the artificial joint capsule of revision hip arthroplasties. *J. Bone Joint. Surg.*, **743**, 57-62.

ATHANASOU, N.A., QUINN, J., FERGUSON, D. & McGEE, J.O.'D. (1991). Bone resorption by macrophage polykaryons of a giant cell tumour of tendon sheath. *Br. J. Cancer*, **63**, 527.

ATHANASOU, N.A., WELLS, C.A., QUINN, J., FERGUSON, D.J.P., HERYET, A. & McGEE, J.O.'D. (1989). The origin and nature of stromal osteoclast-like giant cells in breast carcinoma: implications for tumour osteolysis and macrophage biology. *Br. J. Cancer*, **59**, 491.

CHAMBERS, T.J. & MAGNUS, C.J. (1982). Calcitonin alters behaviour of isolated osteoclasts. *J. Pathol.*, **136**, 27.

CHAMBERS, T.J., REVELL, P.A., FULLER, K. & ATHANASOU, N.A. (1984). Resorption of bone by isolated rabbit osteoclasts. *J. Cell Sci.*, **66**, 383.

EILON, G. & MUNDY, G.R. (1978). Direct resorption of bone by human breast cancer cells *in vitro*. *Nature*, **276**, 726.

FALLON, M.D., TEITELBAUM, S.L. & KAHN, A.J. (1983). Multinucleation enhances macrophage mediated bone resorption. *Lab. Invest.*, **49**, 159.

VAN FURTH, R. (1989). Origin and turnover of monocytes and macrophages. *Current Topics Pathol.*, **79**, 125.

GALASKO, C.S.B. (1976). Mechanisms of bone destruction in the development of skeletal metastases. *Nature*, **263**, 507.

GALASKO, C.S.B. (1982). Mechanisms of lytic and blastic metastatic disease of bone. *Clin. Orthop. Rel. Res.*, **169**, 20.

GOLDE, D.W. & TAKAKU, F. (eds). (1988). *Haemopoietic Stem Cells*. Marcel Dekker: New York.

HASKILL, S. (1981). Collection of macrophages from tumours. In *Manual of Macrophage Methodology: Collection, Characterization and Function*. Herscowitz, N.B., Holden, H.T., Bellanti, J.A., Ghaffer, A. (eds), Marcel Dekker: New York. pp. 43.

KOEFFLER, H.P., MUNDY, G.R., GOLDE, D.W. & CLINE, M.J. (1978). Production of bone resorbing activity in poorly differentiated monocytic malignancy. *Cancer*, **41**, 2438.

MCGEE, M.P. & MYRVIK, Q.N. (1981). Collection of alveolar macrophages from rabbit lungs. In *Manual of Macrophage Methodology: Collection, Characterization and Function*. Herscowitz, N.B., Holden, H.T., Bellanti, J.A., & Ghaffer, A. (eds), Marcel Dekker, New York. pp. 17.

METCALF, D. (1984). Macrophage heterogeneity. In *Mononuclear Phagocyte Biology*. Volknam, A. (ed.), Marcel Dekker: New York. pp. 489.

MINKIN, C. (1982). Bone acid phosphatase: tartrate resistant acid phosphatase as a marker of osteoclast function. *Calcif. Tissue Int.*, **34**, 285.

MUNDY, G.R., ALTMAN, A.J., GONDEK, M.D. & BANDELIN, J.G. (1977). Direct resorption of bone by human monocytes. *Science*, **196**, 1109.

NOVAK, J.F., FUKOSHIMA, H., MCMASTER, J.H., ASANUMA, K. & SIMPSON, K.A. (1984). Bone resorption in osteogenic sarcoma – II, resorption of the bone collagenous matrix by tumor cells, normal fibroblasts and macrophages. *Eur. J. Cancer Clin. Oncol.*, **20**, 939.

TEITELBAUM, S.L., STEWART, C.C. & KAHN, A.J. (1979). Rodent peritoneal macrophages as bone resorbing cells. *Calcif. Tissue Int.*, **27**, 255.

UDAGAWA, N., TAKAHASHI, N., AKATSU, T. & 6 others (1990). Origin of osteoclasts: mature monocytes and macrophages are capable of differentiating into osteoclasts under a suitable microenvironment prepared by bone marrow-derived stromal cells. *PNAS*, **87**, 7260.